# Editorial: Natural products and their derivatives in the treatment of inflammatory and autoimmune diseases

**DOI:** 10.3389/fimmu.2025.1613013

**Published:** 2025-05-14

**Authors:** Mohamed El-Shazly, Jiao Xiao, Jian Chen

**Affiliations:** ^1^ Department of Pharmacognosy, Faculty of Pharmacy, Ain-Shams University, Cairo, Egypt; ^2^ School of Traditional Chinese Materia Medica, Shenyang Pharmaceutical University, Shenyang, China; ^3^ Department of Rheumatism and Immunology, Peking University Shenzhen Hospital, Shenzhen, China; ^4^ Institute of Immunology and Inflammatory Diseases, Shenzhen Peking University-The Hong Kong University of Science and Technology Medical Center, Shenzhen, China; ^5^ Shenzhen Key Laboratory of Inflammatory and Immunology Diseases, Shenzhen, China

**Keywords:** natural products, inflammatory diseases, autoimmune diseases, derivatives, treatment

Inflammatory and autoimmune diseases such as psoriasis, inflammatory bowel disease (IBD), multiple sclerosis (MS), ankylosing spondylitis (AS), systemic lupus erythematosus (SLE), IgA nephropathy (IgAN), rheumatoid arthritis (RA), et al. represent a significant and growing burden on global health (1). Characterized by dysregulated immune responses, these conditions pose serious threats to human well-being and longevity. While decades of intensive research have illuminated the intricate molecular mechanisms underlying these pathologies, leading to the development of various therapeutic interventions, the persistent challenges of drug resistance and adverse side effects underscore the urgent need for innovative therapeutic strategies.

In this context, the realm of natural products and their derivatives emerges as a treasure trove of potential solutions. For centuries, traditional medicine systems have harnessed the power of nature to alleviate a myriad of ailments, and modern science is increasingly validating these time-honored practices. Natural products, particularly their structurally diverse monomer compounds, exhibit a remarkable array of biological activities, positioning them as invaluable sources of novel drugs and promising lead compounds for innovative drug development.

Over recent decades, researchers have achieved significant breakthroughs across chemical sciences, pharmaceutical resources, pharmacological studies, formulation technologies, and novel drug discovery. The remarkable story of Professor Tu You-You and her Nobel Prize-winning discovery of artemisinin serves as a powerful testament to the transformative potential of natural product research. Initially identified for its anti-malarial properties, artemisinin and its analogs have since demonstrated a broader spectrum of activities, including compelling anti-inflammatory and immunomodulatory effects with promising applications in the treatment of immune-related disorders (2). This success story underscores the vast, yet largely untapped, potential residing within the natural world for addressing complex human diseases.

This Research Topic titled “*Natural products and their derivatives in the treatment of inflammatory and autoimmune diseases*” aims to highlight the exciting advancements in this critical area of research. We have gathered a Research Topic of insightful perspectives, cutting-edge original research articles, comprehensive reviews, and thought-provoking commentaries that delve into the functional implications of natural products and their derivatives in combating the intricate challenges of inflammatory and autoimmune conditions.

The contributions within this Research Topic showcase the breadth and depth of ongoing investigations. From comprehensive reviews exploring the multifaceted therapeutic potential of compounds like artemisinin in rheumatic and autoimmune disorders to preclinical studies evaluating the immunomodulatory effects of herbal medicines in neurodegenerative conditions such as amyotrophic lateral sclerosis (ALS), the articles presented here underscore the diverse applications of natural products (Long et al.). Furthermore, investigations into the quality and potential biases in clinical trials of traditional Chinese medicine for MS highlight the importance of rigorous scientific evaluation in this field (Wu et al.).

Several articles within this Research Topic delve into the specific molecular mechanisms by which natural products exert their beneficial effects. Studies targeting post-stroke neuroinflammation with Salvianolic acid A and exploring the anti-inflammatory potential of phenolic acids from medicinal plants exemplify this mechanistic focus (Yang et al.; Xie et al.). In addition, Zheng’s group reported that Chinese herbal medicine represented the potential for the treatment of ALS (Yang et al.). Moreover, investigations into the effects of oral curcumin in models of campylobacteriosis and the immuno-modulatory role of baicalin in atherosclerosis further illustrate the diverse therapeutic targets and mechanisms of action of natural compounds (Heimesaat et al.; Wang et al.). Furthermore, Chinese medicine PaBing-II showed a notable protective effect on human induced pluripotent stem cell (iPSC)-derived dopaminergic neurons (Wu et al.). Beyond direct therapeutic applications, this Research Topic also emphasizes the crucial role of cutting-edge methodologies in advancing the field. The application of network pharmacology and genetic sequencing for identifying potential biomarkers and novel drug targets in inflammatory and autoimmune diseases holds immense promise for developing more precise and effective treatments.

The papers presented in this Research Topic underscores the significant progress being made in harnessing the power of natural products to address the persistent challenges of inflammatory and autoimmune diseases. We are confident that the research highlighted herein will not only contribute to a deeper understanding of the therapeutic potential of natural compounds but will also inspire further investigation and ultimately pave the way for the discovery of novel drugs with independent intellectual property rights, offering new hope for patients suffering from these debilitating conditions.

We extend our sincere gratitude to all the contributing authors for their valuable insights and rigorous research, and to the reviewers for their dedication and expertise in ensuring the high quality of this Research Topic. We hope that this Research Topic will serve as a valuable resource for researchers, clinicians, and anyone interested in the exciting and ever-evolving field of natural product-based therapeutics for inflammatory and autoimmune diseases.
